# Impact of UV-C on material degradation: a scoping literature review

**DOI:** 10.1017/ash.2025.10114

**Published:** 2025-09-02

**Authors:** Daniel Suh, Stacey Hockett Sherlock, Kimberly C. Dukes, Eli N. Perencevich, Alexandre R. Marra

**Affiliations:** 1 Center for Access and Delivery Research and Evaluation (CADRE), Iowa City VA Healthcare System, Iowa City, IA, USA; 2 Department of Internal Medicine, Carver College of Medicine, University of Iowa, Iowa City, Iowa, USA; 3 Faculdade Israelita de Ciências da Saúde Albert Einstein, Hospital Israelita Albert Einstein, São Paulo, SP, Brazil

## Abstract

**Background:**

Ultraviolet-C (UV-C) radiation has emerged as a widely adopted disinfection technology in healthcare settings due to its germicidal effectiveness. However, concerns have grown regarding the potential degradation of materials, particularly polymeric surfaces, subjected to repeated UV-C exposure. Understanding the extent, mechanism, and contributing factors of UV-C-induced material degradation is essential for safe and sustainable implementation.

**Methods:**

We conducted a scoping literature review in accordance with PRISMA guidelines to evaluate evidence on UV-C-related material degradation. Multiple databases were searched for studies published between January 1, 2000, and August 30, 2024, investigating material degradation under UV-C radiation (100–280 nm) in potentially healthcare-relevant conditions. Data abstraction captured study design, UV-C exposure characteristics, material types, degradation types, and assessment methods.

**Results:**

Of the 56 studies reviewed, 14 met inclusion criteria. All employed experimental designs conducted in laboratory settings. UV-C exposure resulted in both visible and structural degradation of several polymeric materials. Polycarbonate, HDPE, and PLA were the most affected, exhibiting yellowing, surface cracking, and loss of mechanical strength. Degradation was time-, dose-, and distance-dependent, with longer exposure, higher irradiance, and shorter distance correlating with more severe damage. Detection methods included visual inspection, microscopy, spectroscopy, and nanoindentation. Some studies reported UV stabilizers and antioxidant additives as potential mitigation strategies.

**Conclusions:**

UV-C radiation can cause significant degradation of commonly used polymeric materials. These findings underscore the need for careful selection of materials in UV-C environments and support further research on mitigation strategies to enhance material longevity.

## Introduction

In healthcare environments, the importance of maintaining a sterile and pathogen-free environment is paramount.^1^ Ultraviolet-C (UV-C) radiation, spanning wavelengths between 100 and 280 nanometers, has emerged as a compelling tool in diverse applications, particularly within healthcare settings where stringent disinfection measures are crucial.^1,2^

For example, a large-scale evaluation of UV-C disinfection technology in the Veterans Health Administration found an associated 19% reduction in incidence of Gram-negative bacteria after implementation of the systems in 40 acute care hospitals.^3^ However, UV-C disinfection has yielded conflicting results in reducing multidrug-resistant organisms.^4,5^

UV-C radiation’s ability to induce photochemical reactions and degrade material presents an intriguing avenue for enhancing disinfection practices.^6^ The potential benefits of harnessing UV-C for decontamination purposes have prompted investigations into the potential secondary negative impact on different types of materials.^7^ Understanding the impact of UV-C exposure on materials used in healthcare settings is essential for ensuring the safety, efficacy, and longevity of medical equipment and surfaces subjected to UV-C disinfection protocols.^2,8^

Against this backdrop, a scoping literature review becomes imperative to consolidate and critically evaluate existing knowledge regarding the influence of UV-C on material degradation.^2,7^ Hence, our objective was to conduct a literature review to synthesize findings from a range of studies, exploring the diverse effects of UV exposure on various materials, including polymers, metals, and composites. Through this scoping literature review, we aim to contribute insights that may inform future research directions and practical applications of UV-C-induced material degradation within healthcare settings.

## Methods

### Scoping literature review and inclusion and exclusion criteria

This scoping literature review focused on UV-C material degradation and was conducted according to the Preferred Reporting Items for Systematic Reviews and Meta-Analysis (PRISMA) statement.^9^ Institutional Review Board approval was not required. Inclusion criteria for studies in this scoping review were as follows: 1) original research manuscripts, studies presented at scientific conferences (eg, abstracts or proceedings), dissertations, and theses; 2) published in peer-reviewed, scientific journals; 3) involving material that may be exposed to UV-C radiation in a typical potential hospital environment; and 4) utilizing observational or experimental study designs. UV-C exposure refers to the act of subjecting a material to UV radiation in the short-wave UV-C spectrum, typically with wavelengths between 100 and 280 nanometers.^8^ Material degradation was defined as the process by which a material undergoes physical, chemical, or structural deterioration, often resulting in a decrease in its original properties or performance.^7^ The literature search included studies published or presented from January 1, 2000 to August 30, 2024. Exclusion criteria comprised editorials, commentaries, reviews, study protocols, and studies analyzing topics beyond UV-C material degradation. Within the category of studies analyzing UV-C material degradation, we excluded studies of UV-C degradation of materials for simulated outdoor environments, articles examining UV-C degradation of portable hospital equipment or medical devices (eg, endoscopes), and the exclusive examination of microplastics.

### Search strategy

**Figure 1.**
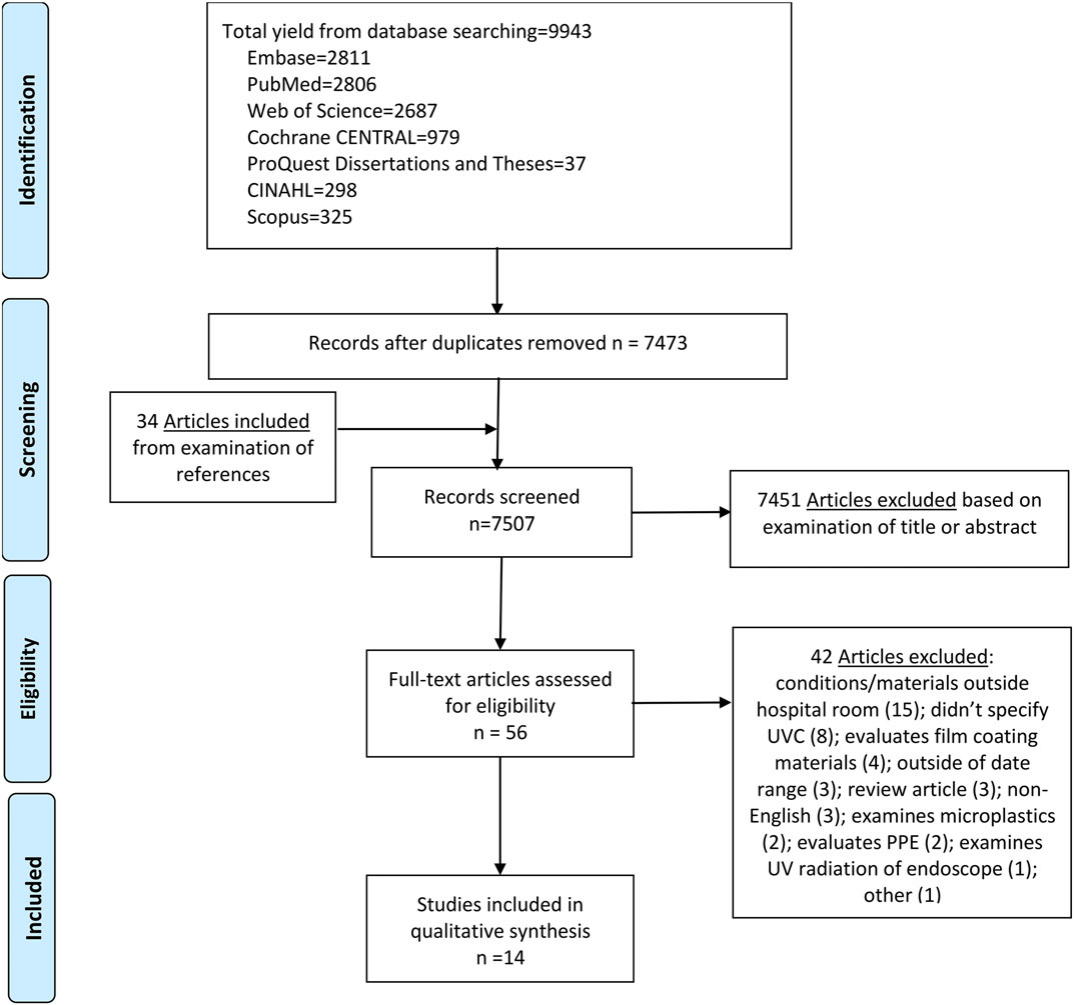
Literature search for articles on the impact of UV-C exposure on material degradation.

We performed literature searches in PubMed, Cumulative Index to Nursing and Allied Health (CINAHL), Embase (Elsevier Platform), Cochrane Central Register of Controlled Trials, Scopus (which includes EMBASE abstracts), Web of Science, and ProQuest Dissertations and Theses. The entire search strategy is described in Supplementary Appendix 1. This study uses the PICO framework,^10^ focusing on materials used in healthcare settings (P) and exposure to UV-C radiation (100–280 nanometers) (I), with the comparison not explicitly stated but implicitly made to materials not exposed to UV-C radiation or to baseline conditions before UV-C exposure (C). The primary outcome of interest (O) was to detect any physical, chemical, or structural degradation of materials resulting in decreased performance properties. After applying the exclusion criteria, we reviewed 56 papers, out of which 14 met the inclusion criteria and were included in the scoping literature review (Figure 1).

### Data abstraction

Titles and abstracts of all articles were screened to assess whether they met the inclusion criteria. Abstract screening was performed by two reviewers (DS and ARM). Of four independent reviewers (DS, SMHS, KCD, and ARM), two independently abstracted data for each article using a standardized abstraction form. Reviewers resolved disagreements by consensus.

The reviewers abstracted data on study design, location, time of UV-C exposure (min/hours), characteristics of UV-C exposure, type of material reported to UV-C degradation, method to detect the surface or material changes due to UV-C exposure, type of degradation detected on the surface or material changes, and conclusions if available.

## Results

### Characteristics of included studies

Of 56 studies reviewed in further detail, 14 met the inclusion criteria for this scoping literature review.^11–24^ All included articles were experimental designs carried out in laboratories rather than field environments. These studies primarily used UV-C radiation exposure in controlled laboratory settings to simulate material degradation under various conditions. Notably, these studies provided quantifiable data on exposure times (ranging from 1 h to 2 700 h), irradiance levels (from 52.5 µW/cm^2^ to 12,000 µW/cm^2^), and source distances (5 cm–1.5 m), offering concrete thresholds for material degradation.

### Documented effects of UV-C exposure on polymeric materials

The documented effects of UV-C exposure on polymeric materials (Table 1) were consistent across multiple studies.^11–17,20–24^ A common effect was color change, with materials such as polycarbonate and high-density polyethylene (HDPE) exhibiting significant yellowing and a loss of transparency when exposed to UV-C radiation. Surface cracking and microfractures were also widely reported, especially in plastics like polycarbonate, where cellular cracking became more pronounced as exposure time increased. Quantitative analysis revealed that polycarbonate exposed to 88 µW/cm^2^ at 1.5 m showed measurable yellowing within 72 h, while HDPE developed surface cracks after 144 h at similar irradiance.^20^ Mechanical property degradation was a common result, with many materials, including polycarbonate and polylactic acid (PLA), showing reduced stress and strain at break.

**Table 1.** Examples of polymeric and non-polymeric materials

Polymeric materials	Non-polymeric materials
**Polyvinyl chloride (PVC):** IV tubing, blood bags	**Stainless steel:** Surgical instruments, implants
**Polypropylene:** Syringes, disposable lab ware	**Glass:** Vials, lab equipment
**Polycarbonate:** Medical device housings (patient monitors, infusion pumps, ventilator components, dialysis machines)	**Titanium:** Dental/orthopedic implants
**Polyethylene:** Sterile containers, tubing connectors	**Ceramics:** Dental crowns, bone grafts
**Polylactic acid (PLA):** 3D printed implants	**Natural rubber (latex):** Gloves, elastic bandages
**Polyurethane:** Wound dressings, hospital mattress coatings	**Aluminum:** Wheelchair part, portable device frames

### Time-dependent degradation

UV-C degradation was consistently found to be time-dependent across various materials.^20,23,24^ Polycarbonate, for instance, exhibited progressive mechanical and molecular changes over 72–216 h of UV-C exposure. The material’s stress at break and strain at break decreased over time, with more severe reductions occurring at longer exposure times.^20^ Similarly, polylactic acid (PLA) demonstrated a reduction in tensile and compressive strength, with the most significant changes occurring after 24 h of exposure.^24^ Other materials like polyethylene terephthalate (PET) and high-density polyethylene (HDPE) also displayed substantial degradation over extended periods of UV-C exposure, indicating that prolonged exposure leads to more significant material damage.^23^

### Dose-response relationship between the intensity of UV-C irradiation and the degree of material degradation

A clear dose-response relationship was observed between UV-C intensity and the degree of material degradation.^12,13^ Materials exposed to higher UV-C irradiance levels exhibited increased surface degradation, such as deeper craters and greater surface roughness.^20,23^ Heating, ventilation, and air conditioning (HVAC) components exposed to >1,000 µW/cm^2^ showed accelerated mass loss,^15^ while polycarbonate developed surface craters 2–3 times deeper at 12,000 µW/cm^2^ compared to 310 µW/cm^2^.^23^ These quantitative relationships emphasize the need for careful irradiance control in clinical settings.

### Distance-dependent degradation patterns

Several studies systematically evaluated distance effects on UV-C degradation. At close ranges (5–50 cm), materials showed accelerated damage—polycarbonate developed 2.1 times faster yellowing at 50 cm versus 1.5 m,^20^ while PLA exhibited 13% greater tensile loss.^12^ Polyvinyl chloride (PVC) demonstrated cytotoxic effects only at ≤50 cm distances.^14^ Only acrylonitrile butadiene styrene-polycarbonate copolymer (ABS-PC) blends maintained stability during 24-hour exposures in irradiation chambers.^13^

### Impact of material properties

The impact of UV-C exposure on material properties varied depending on the type of material.^12–15,17,19,20^ The impact appears to be material-dependent. Color changes were the most common visible effect, with materials such as polycarbonate and high-density polyethylene (HDPE) undergoing significant yellowing.^12,17,20^ Surface cracks and microfractures were also frequently observed, particularly in polycarbonate and polylactic acid (PLA). In terms of mechanical properties, several materials exhibited reduced stress and strain at break after UV-C exposure, suggesting that UV radiation weakened the molecular structure, making them more fragile.^12–15,19,20^ While the color change and fading were often considered aesthetic issues, these changes did not always correlate with a loss of functional properties.^17^ Processing of materials (e.g. method of 3D printing, veneer production method) was found to affect the degradation process.^12^

### Methods to detect material degradation exposed to UV-C radiation

Various methods were employed to detect material degradation caused by UV-C exposure.^20,24^ Visual inspection was a common technique used across studies to identify color changes and surface damage, such as cracks and discoloration. Microscopic methods, such as optical microscopy and scanning electron microscopy (SEM), were frequently used to examine the finer details of surface degradation, such as microfractures, roughness, and changes in the microstructure of the materials.^20,24^ Infrared spectroscopy was another key method used to detect molecular changes in the materials, such as bond dissociation and chain reorganization, which occurred as a result of UV-C exposure.^22^ Additionally, nanoindentation was utilized to measure changes in material hardness, helping to assess whether a material became more brittle or softened after UV exposure.^22^

### Mitigation strategies

Some studies discussed potential mitigation strategies to reduce the effects of UV-C degradation.^12,18^ The use of additives, such as UV stabilizers and antioxidants, was suggested as a method to slow down the degradation process in materials like polycarbonate and PLA.^12,18^

## Discussion

This scoping review examined the available literature on the degradation of polymeric materials following UV-C radiation exposure, with particular focus on hospital-relevant contexts. While UV-C technology is increasingly adopted for disinfection purposes in healthcare settings, the collateral impact on material, especially polymers, warrants thorough understanding to guide evidence-based procurement and maintenance strategies including cost-effectiveness analysis.

The literature consistently demonstrates that certain polymeric material are more susceptible to UV-C-induced degradation.^11–24^ Importantly, three key factors emerged: exposure time, irradiance dose, and distance from UV-C sources (Table 2). Polycarbonate and high-density polyethylene (HDPE), for instance, showed considerable vulnerability, including pronounced yellowing, surface cracking, and reduction in mechanical integrity.^13,20,23^ These effects were evident across a range of exposure times (24–72 h for significant polymer degradation)^12,13,20^ and intensities (e.g. >1,000 µW/cm^2^ for HVAC components),^15^ underscoring the material-specific nature of UV-C damage. Polymeric materials such as polyactic acid (PLA) also demonstrated substantial performance loss, particularly in tensile strength and elasticity.^12,24^

**Table 2.** Summary of characteristics of studies included in the scoping literature review

First author, year, location Study design Exposure time	Characteristics of UV-C exposure	Type of material	Method to detect the surface or material changes	Type of degradation detected	Key findings
Aboamer et al.^11^, Riyadh, Saudi Arabia Experimental 1 hour	254 nm, 20W lamps, 8–16 cm distance Custom chamber (610 × 152 × 108 mm, 4.5 kg) ILT radiometer (irradiance) Wireless loggers (temp/RH)	*Polymeric materials:* epoxy resin and powdered neodymium–iron–boron magnet (NdFeB) mixed at 1:1 ratio; 20 specimens manufactured; 10 for tensile test and 10 for compression test	Microscopic inspection Tensile and compression tests (ASTM D3039/ D3410), respectively Energy dispersive spectroscopy (EDS)	Property changes: Physical: Surface cracks/fissures formation - Chemical: Corrosion development Tensile: Transition force increased from .41 kN to .58 kN postUV-C Compression: Yield force rose from 4.9 kN to 6.0 kN, indicating greater compressive strength gain versus tensile – EDS: Carbon content increased from 71.69% to 78.56%, suggesting material hardening	Study focus - Compare pre /postUV-C stress-strain curves to assess disinfection impact on magnetic polymer composites - Findings confirm UV-C enhances mechanical/chemical properties, supporting clinical use
Amza et al. ^12^, Romania Experimental Study 24 h	-BS-02 irradiation chamber (Opsystec Dr. Grobel (Ettlingen, Germany) 24-hour UV-C exposure aging cycle (then left to cool to room temp for 4 h) vs control group; 24 hour cycle meant to simulate repeated UV-C procedures	*Polymeric materials:* Materials 3D printed from common polymers, polylactic acid (PLA) and polyethylene terephthalate-glycol (PETG)	Microscopic inspection—Scanning Electron Microscopy (Quanta Inspect F50) SEM (scanning electron microscope) Rig made to exert constant tensile force for 5 days, with dimensional inspection and measurement at different intervals and elongation, and measurement of material creep compliance by measuring torque with a torque sensor	Color changes: none except darkening and yellowing of the transparent PETG samples Physical property changes: no significant impact on part dimensions (for either part geometry or print orientation); for tensile strength, fracture modes were similar for controls and irradiated samples, with a 9.1% tensile strength loss for PLA and 38.1% loss for PETG after irradiation; no significant change in stiffness for PLA and 5.1% reduction in Young’s Modulus for PETG; for compressive strength, 45% loss	-Study evaluated UV-C effects on 3D printed polymers: -PLA: 7–8% strength loss -PETG: >30% degradation (not recommended for UV-C exposure) -Reduced creep resistance in both materials -Key implications: -MEX 3D printing method affects UV-C resistance -Caution for long-term stressed components -UV-resistant PETG blends recommended -In-house testing advised preproduction
Amza et al.^13^, Romania Experimental 24 h	UV-C bulb at 254 nm and UV-B at 315 nm Opsytec Dr. Grobel BS-02 irradiation chamber (Ettlingen, Germany) Samples exposed to 254 nm radiation for 24 h at a temperature of 50°C and a radiating power of 10 W/m2, left in the irradiation chamber for an additional 4 h to cool down to room temperature; control group (non-irradiated) compared to sample groups that were exposed to UV-C only and UV-B only	*Polymeric materials*:- 3D-printed Z-PCABS blend (Zortrax, Olsztyn, Poland) - Composition: 55–60% ABS, 30–35% PC, ≤10% additives/colorants - Thermal properties: Tg = 104°C, Tm = 260°C - Mechanical properties: Tensile strength = 36.89 MPa, Density = 1.14 g/cm^3^	tensile strength (using Instron 8 872 machine), compressive strength (using Instron 8 801 machine), and mechanical property tests Tensile load was measured after 2h, 6h, 21h, and 24h then once ever 24h for a total of 168h (7 days)	Physical property changes:1.86% lower tensile strength compared to control samples (38.53 MPa vs 39.26 Mpa) which was not statistically significant (F = 1.91, *P* = .20). On average, the Young’s Modulus of aged samples decreased by 5.51% vs control samples (2075MPa vs 2196MPa) which was determined to be statistically significant (F = 5.70, *P* = .04). 6.5% lower compressive strength compared to control group (57.82 MPa vs 61.81 MPa; F—36.3, *P* = 3 × 10–4)	This UV-C accelerated aging study on MEX 3D-printed ABS-PC copolymer at 24-hour exposure showed: tensile strength maintained (no significant change vs control), minor reductions in stiffness (−5.2%) and compressive strength (−6.5%) Creep behavior remained unaffected. ABS-PC demonstrates excellent UV-C stability, making it suitable for UV-sterilized applications.
Haishima et al.^14^, Japan Experimental Study Varied by condition: (1) 4.5 hrs, (2) 25 days, (3) 1 month	-Condition 1: UV germicidal lamp, 52.5 µW/cm,^2^ 136 J/cm^2^ -Condition 2: 254-nm UV lamp (Model ENF-260C/J; Spectronics Corporation, NY), .45 mW/cm,^2^ 972 J/cm^2^ -Condition 3: 254-nm UV lamp with filter (TG-100C, 100W grid tube, 50/60 Hz, Vilber Lourmat; Marne-la-Vall_ee Cedex 1, France), 8.3 mW/cm,^2^ 134 J/cm^2^ -Different intensities and exposure times tested	*Polymeric materials*: polyvinyl chloride (PVC) sheets; medical-grade PVC sheets (thickness 1/4 .4 mm, total contents of DEHP and MEHP 1/4 28.7%, and .10 w/w). DEHP and PVC sheets containing no additives or DEHP alone were also provided	Microscopic inspection Tensile/contact angle: UV-PVC ≈ control -GC-MS: DEHP/MEHP-Me (120°C→300°C, BPX-5 column) -XPS/FT-IR: Control (C, O, Cl, Si); UV (↑O, ↓Cl, highest oxidation in Cond. 2) -Cytotoxicity (IC_50_): Control (non-toxic); Cond. 1 (22.2%); Cond. 2 (7.8%); Cond. 3 (83.1%) -CA test: Control (negative); Cond. 1 (positive, dose-dependent); Cond. 3 (negative)	Color changes: slight yellowing but transparency maintained suppression of DEHP elution under specific conditions	The study found that UV irradiation can modify the surface of PVC medical devices to suppress the elution of DEHP plasticizers. However, some UV conditions led to toxicity due to DEHP oxidation products. The safest approach was a short, high-intensity UV-C exposure (condition 3: 8.3 mW/cm,^2^ 134 J/cm^2^), followed by methanol washing and gamma sterilization, which effectively reduced DEHP elution and prevented toxicity.
Kauffman and Wolf^15^, Dayton, OH Experimental 2 700 h	- Duration varied per material UV-C (254 nm) exposure at 1 000, 4 000, 7 000, 11 000 μW/cm^2^ (max 2 700 h) -Test termination based on sample-specific analytical results -Temperature-controlled at 25°C (77 °F) -LZC-ICH2 photoreactor with 8 lamps and rotating platform (6 rpm) -Irradiance adjusted using screen between lamps and platform	*Polymeric materials:* --polymers containing fillers --other sealants, filters, filter media, and other components used in HVAC systems -elastomers and sealants *Non-polymeric materials:* --organic foam and fibrous materials	Visual inspection: Naked-eye observation combined with SEM/EDS surface analysis Microscopic examination: Detailed surface characterization - Material-specific evaluations: Elastomers tested for surface elasticity (TMA); polished polymers analyzed for crater formation (profilometer); foams/fibrous materials measured for mass loss (analytical balance) - Data correlation: Mass loss/elasticity changes plotted against exposure time at each irradiance level to identify breakpoints (surface composition changes) and assess independent effects of dosage, time, and irradiance on photodegradation	-Color changes: reported “darkening -Physical property changes: “cracks”; “morphological or compositional changes in the exposed surface”	-Identified HVAC materials unsuitable for unprotected UV-C exposure -Solid polymer films (slightly dulled) resist UV-C better than porous foams/meshes -Additives may accelerate degradation; woven glass fibers improve UV-C resistance -Results vary by manufacturer (materials/processing differences) -Lab testing under HVAC-specific UV-C/temperature required for accurate lifespan
Liu et al.^16^, Beijing, China; Auburn, AL Experimental Study 0–100 h	254/313/340/365/420 nm wavelengths at 0/5/10/20/40/60/100 h intervals Ultraviolet accelerated aging box (Zinc-P, Beijing North Li-fang Co.) Black panel thermometer 55°C Relative humidity 50 ± 2% Irradiance 35 W/m^2^	*Non-polymeric materials:* --air dried Asian White Birch sapwood veneer (Betula playphylla Suk.) with Azo Acid Red GR (C. I. Acid Red 73) dye and Acid Blue V (C. I. Acid Blue 1).	Color measurements: CIE L * a * b * system (Datacolor SF600 Plus-CT) with D65 illuminant/10° observer for ΔE Reflectance analysis: Converted to K/S spectra via Kubelka-Munk equation Chemical characterization: ATR-FTIR (Nicolet Nexus 670, diamond crystal, 4 000–400 cm^−1^ range, 8 cm^−1^ resolution, 64 scans)	Color changes: ABV/ARG-dyed wood showed progressive fading (complete at 100 h). 254 nm caused fastest initial change (20 h). Undyed birch: intermediate between ARG (stable) and ABV (unstable). Reflectance: ABV reflectance shifted most (490–600 nm). Lignin degradation: Varied from 254 nm (worst) to 420 nm (mildest).	Examined ABV/ARG veneers under 254–420 nm UV. ARG > ABV stability. 254 nm caused rapid initial change (20 h); 313/340 nm induced greater long-term discoloration. Lignin/dye degradation linked to wavelength/duration. Solutions: lightfast dyes, UV-absorbing coatings
Mitxelena-Iribarren et al.^17^, Spain Experimental Study Equivalent of 16 years	-Exposure times: 3.5h (1yr), 7h (2yr), 14h (4yr), 28h (8yr), 54h (16yr) 36.8 mJ/cm^2^ per treatment 1 m from lamp 254 nm Lamp: Zenzoe (ASTI MOBILE ROBOTICS/Boos) - 5 × 254 nm tubes, 140W total Sample preparation: -Colorimetry/microstructure: 3 × 3 cm samples -Tensile tests: 1 × 10 cm samples -Experimental design: -≥3 replicates per material/size -Control samples for all materials	*Polymeric materials*: --Fabrics --Polymers *Non-polymeric materials*: --Woods --Metals --Stainless steel	Visual inspection Microscopic inspection: Phenom G2 26 PRO SEM surface imaging ImageJ analysis: --Fibrous materials: 20 fiber width measurements --Non-fibrous: Grey-intensity roughness analysis Colorimetry: -RGB model quantification via Matlab Tensile testing: (Zwikiline 1.0 machine (5–50N range), testXpert III control software	-Color changes: -Polymers: yellowing -Fabrics: color fading (blue/green affected > red) -Uncoated MDF: measurable but not visible change -Stainless steel: oxidized after 8-year equivalent dose Physical changes: -Surface polishing effect (reduced roughness) -Polymers/woods: significant surface flattening Mechanical properties: -No tensile strength change at 1-year equivalent dose -16-year dose: reduced max load (cottons/polyesters most affected) -SEM findings: -No fiber diameter changes in colored materials -Surface microstructure altered in PP, varnished MDF, metals -Rough materials showed significant smoothness increase	UV-C alters appearance but preserves mechanical functionality. Changes include: -Polymers: yellowing -Fabrics: color fading (varies by dye) -Non-fibrous materials: significant surface microstructure changes -Woods/metals: no discoloration -Tensile strength unaffected in all materials -Conclusion: UV-C impacts aesthetics only, not structural integrity
Olewnik-Kruszkowska et al.^18^, Poland Experimental Study 2–16 h	254 nm low-pressure mercury vapor lamp (TUV30W, Philips) 5 cm distance (3.12 mW/cm^2^ intensity) 2–16 h exposure (230–1840 kJ/m^2^ doses) room temperature, air atmosphere complete degradation after 16 h	*Polymeric materials:* Polylactide (2002D, NatureWorks), MFR 5–7 g/10 min (2.16 kg, 190°C), density 1.24 g/cm^3^, Mw ∼ 155,500 Da, D/L content 3.5/96.5% – montmorillonite K-10 (Acros Organics) as nanofiller—poly(ε-caprolactone) (PCL, CAPA 6 506, Solvay), Mw 50,000 g/mol as compatibilizer	Gel permeation chromatography (GPC) and 1H nuclear magnetic resonance (1H NMR) techniques Fourier transfer infrared spectroscopy (FITR)	FTIR analysis indicated that montmorillonite incorporation delayed UV-C degradation, contrasting with most literature reports of nanofiller-accelerated degradation. GPC results confirmed this retardation effect, showing gradual molecular weight decrease with increasing clay content. PCL was found to promote polymer breakdown, facilitating conversion of high to low molecular weight chains.	-Complete degradation after 16h UV-C (254 nm) exposure -Montmorillonite delays degradation rate -1H NMR/GPC results: --Polylactide chain scission → low MW species --Neat PLA degrades faster than composites -Kinetics altered by: --Montmorillonite addition --Nanoclay (± compatibilizer)
Rainer et al.^19^, Rome, Italy Experimental Study NR	-Distance/s from UV-C exposure: Not explicitly stated -UV-C nm wavelength/s: Not explicitly stated UV irradiation as a sterilization technique - UV-C was tested alongside autoclave, ethanol soaking, hydrogen peroxide gas plasma (HPGP), and dry oven sterilization for comparative effectiveness	*Polymeric materials:* Electrospun poly-L-lactide (PLLA) scaffolds	Visual inspection (naked eye, without magnification) Microscopic inspection - Field Emission Scanning Electron Microscopy - FE-SEM Attenuated Total Reflectance Fourier Transform Infrared Spectroscopy (ATR-FTIR) and Differential Scanning Calorimetry (DSC)	Physical property changes: -Slight decrease in crystallinity of PLLA scaffolds. -No significant chemical changes were detected via ATR-FTIR.	-UV-C irradiation effective for sterilizing PLLA scaffolds -UV-C preserved PLLA chemical composition vs autoclaving/oven treatments -Minor crystallinity decrease observed -Recommended as non-destructive sterilization method -Suitable for biomedical applications (effective + minimal polymer impact)
Redjala et al.^20^, Blois Cedex, France; Algeria Experimental Study 72, 144, 216 h	254 nm, 1.5 m, 88 µW/cm^2^ 2 x Philips T8 25 W lamps mounted in parallel under its vault - Aging times were 72, 114, and 216 hrs	*Polymeric materials:* Polycarbonate	Visual inspection: naked eye evaluation Microscopic inspection: OM/SEM imaging -Analytical methods: --FTIR spectroscopy --UV-Vis spectrophotometry --X-ray diffraction -Mechanical testing: --Microhardness (THV-401 Micro-Vickers tester, 136° diamond indenter) --Tensile tests (MTS Model 43, 10 kN load cell) -Imaging systems: --Olympus BX60 OM (1 000× magnification) --TESCAN MIRA3 SEM (10 kV, 10^−7^ torr vacuum)	Color changes: increased yellowing (↑UV exposure = ↓transparency) Physical changes: --Microhardness affected on both exposed/unexposed sides Tensile properties: ---Stress at break ↓39% (72h), ↓47% (216h) ---Strain at break ↓61% (72h), ↓64% (216h) --Cellular cracking (OM/SEM): worsens with exposure time Structural changes (FTIR): --↓Absorbance at ∼1 000 cm^−1^ (carbonate bond stretching) --Molecular chain dissociation observed	-UV-C effects on material: -Optical: transparency decreased -Chemical: FTIR showed vulnerable bond breakage (↓elasticity) -Structural: XRD revealed crystallite size changes -Mechanical: --Microhardness ↓ through thickness --Tensile tests showed embrittlement (↓properties) -Morphological: --OM/SEM observed cell cracking (stress concentration) --SEM confirmed fragile fracture behavior -Conclusion: UV-C breaks bonds → molecular reorganization → ↓physical/mechanical properties
Shao et al.^21^, Bejing, China Experimental Study 100 h	UV-C exposure setup varied 14 cm (veneer to UV tube axis) 254 nm, 313 nm, 340 nm, 365 nm, 420nm 4-tube UV accelerated aging chamber 55°C BPT Color measurement: Intervals: 0h-100h (8 timepoints) Device: DataColor SF600 Plus CT	*Non-polymeric materials:* --air dried Asian White Birch sapwood veneer (B. playphylla Suk.) with Acid Red GR (C. I. Acid Red 73 made in China), Acid Blue V (C. I. Acid Blue 1 made in China)	CIE (1976) L * a * b * color system; the colorimeter device Data Color (SF600 Plus CT) using light source D65, viewing angle 10 degrees; a “computer color matching system” was used to measure color of each sample after 0, 5, 10, 20, 40, 60, 80 and 100 h of irradiation	Color changes: All wavelengths led to observed discoloration of dyed veneer, along with degradation of wood itself and fading dye. The degree of discoloration varied by wavelength. 254 nm (UV-C) rapidly decreased “color stability of dye stuffs.” The dye itself affected the response to UV light exposure; the red showed less effect, e.g., slight yellowing, but the blue faded and became reddish	-Study tested UV wavelength/exposure time effects on dyed veneers: -254 nm UV-C caused rapid color instability (CIE L * a * b * system) -Dye type influenced results: --Red dye showed higher resistance --Blue dye degraded faster at max exposure -Goal: Inform future stabilizer research
Teska et al.^22^, USA Experimental study NR	6.4 h at 1 m distance -Low-pressure mercury bulb (GE G3675) Dosimetry: -GandR Labs 221 UV meter -75 mJ/cm^2^ average accumulated dose Postexposure: -Masking tape removed from controls -Both exposed/unexposed areas characterized	*Polymeric materials:* Ten grades of plastic materials	Visual appearance assessment Chemical composition analysis, surface roughness and hardness measurements using microscopy, spectrophometry, contact angle analysis, infrared spectroscopy, profilometry, and nanoidentification	Chemical damage: 9/10 materials (FTIR detection) Surface microfractures: observed in multiple plastics (OM) Contact angle changes: --Most plastics affected --Polyester/clear acrylic showed greatest change in surface energy Color changes: --Most plastics affected --White acrylic showed most degradation Nanoindentation results: --2 plastics became harder (increased brittleness) --Most became softer	The study followed an experimental design to systematically investigate the impact of UV-C light exposure on plastic materials used in healthcare surfaces, employing various characterization techniques to analyze the effects on the materials’ physicochemical properties
Wolf et al.^23^, USA Experimental 3–6 months	254 nm (spectrum differences LED vs lamps) Exposure systems: 1) LZC-ICH2 photoreactor (8 × LZC-UV-C lamps, rotating platform, temp control) Adjustable irradiance: 310–12,000 µW/cm^2^ via screens 2) Portable LED system (aluminum test cell, 1.5 mm exposure aperture) Exposure durations: 3 and 6 months	*Polymeric materials:* HDPE, polyacetal, PBT, PET (known susceptibility), fiberglass air filter, RTV silicone sealant, Styrofoam (known susceptibility)	Visual inspection Stylus and optical profilometers	Color change Physical property changes: Styrofoam: loss of structural integrity; silicone sealant: loss of flexibility	-Highly UV-C susceptible materials: -polyacetal, HDPE, PBT, PET polymers -silicone sealant, Styrofoam, fiberglass air filter -Crater depth/surface damage correlated with UV-C irradiance level -Degradation monitoring requires further development: -complex light reflection/transmission relationships -needs refinement for LED test setup integration
Zapciu et al.^24^, Romania/Germany/Italy Experimental 24 h	254 nm Opsytec Dr. Grobel BS-02 irradiation chamber (Ettlingen, Germany)	*Polymeric materials:* samples 3D printed from thermoplastic polylactic acid (PLA) by material extrusion process (MEX)	SEM (scanning electron microscope) analysis	Physical property changes: -Tensile strength ↓9% -Compressive strength ↓13% -Flexure ↓2% (↑brittleness) -Changed failure modes (reduced necking/plastic flow)	Tested under standard atmospheric conditions, UV-C had effects on 3D printed PLA. Recommendations: -Consider UV limitations for emergency 3D printing -Test prolonged UV exposure in varied conditions

**Abbreviations:** 1H NMR = proton nuclear magnetic resonance; ABV = Acid Blue V; ABS = acrylonitrile butadiene styrene; ABS-PC = ABS-polycarbonate copolymer; ARG = Acid Red GR; ATR-FTIR = attenuated total reflectance FTIR; BPT = black panel temperature; BPX-5=non-polar GC column; CA = chromosome aberration; CIE L * a * b*=International Commission on Illumination color space; cm^−1^=wavenumber; DEHP = di(2-ethylhexyl) phthalate; EDS = energy-dispersive X-ray spectroscopy; FTIR = Fourier-transform infrared spectroscopy; GC-MS = gas chromatography-mass spectrometry; GE = General Electric; GPC = gel permeation chromatography; GandR Labs 221 UV meter=UVC radiometer; HDPE = high-density polyethylene; h = h; HVAC = heating, ventilation, air conditioning; IC_50_=half-maximal inhibitory concentration; ILT = International Light Technologies; kN=kilonewton; kg=kilograms; kV=kilovolt; LED = light-emitting diode; LZC-ICH2=Luzchem photoreactor; MDF = medium-density fiberboard; MEHP = mono(2-ethylhexyl) phthalate; MEHP-Me=carboxyl-methylated MEHP; MEX = material extrusion 3D printing; MFR = melt flow rate; mm=millimeters; m = meter; MPa=megapascals; MMT = montmorillonite; Mw=molecular weight; mJ/cm^2^=millijoules/cm^2^; μW/cm^2^=microwatts/cm^2^; N = newtons; nm=nanometer; NR = not reported; OM = optical microscopy; PC = polycarbonate; PBT = polybutylene terephthalate; PCL = polycaprolactone; PET = polyethylene terephthalate; PETG = PET glycol-modified; PLA = polylactic acid; PLLA = poly(L-lactic acid); PP = polypropylene; PVC = polyvinyl chloride; RGB = red-green-blue; rpm=revolutions/minute; RT = room temperature; RTV = room-temperature vulcanizing; SEM = scanning electron microscopy; Tg=glass transition temperature; Tm=melting temperature; torr≈1 mmHg; TMA = thermomechanical analyzer; TUV30W=Philips 30W UV-C lamp (254 nm); UV-C=ultraviolet-C; UV-Vis=ultraviolet-visible spectroscopy; W = watts; XPS = X-ray photoelectron spectroscopy; XRD = X-ray diffraction; yr=years; ↓=decreased; ↑=increased; g/cm^3^=grams/cm^3^.

UV-C-associated degradation was clearly shown to be time-dependent. The cumulative exposure to UV-C radiation correlates with a progressive decline in mechanical properties and increased surface and molecular damage. For example, polycarbonate and PLA demonstrated decreased stress at break, microstructural disruption, and more severe physical deformation with longer exposure times.^12,24^ This trend suggests that even intermittent use of UV-C disinfection methods could pose long-term risks to the durability of installed material, particularly in high-turnover clinical environments.

A dose-response relationship was evident in several studies, indicating that higher irradiance or prolonged exposure leads to more pronounced degradation.^12,13,20,23^ This was particularly evident in the degree of surface roughness, crater formation, and chemical bond breakdown observed in materials subjected to high-intensity UV-C doses.^23^ This finding is important in guiding thresholds for UV-C use in clinical setting and emphasizes the importance of dose control when designing disinfection protocols.^6^

These findings suggest that equipment or surfaces consisting of certain polymers undergoing daily UV-C disinfection (typically 15–30 mins per cycle) may reach critical degradation thresholds within months of use. However, importantly, it is not yet clear how the findings from this review apply to materials commonly used in hospitals. It is not known whether any equipment, furnishings, or surfaces typically used in hospitals are manufactured from the specific polymers described in the studies that were reviewed, or whether or to what extent protective coatings or degradation-mitigating manufacturing processes are used in hospital equipment, furnishings, or surfaces. Before applying findings from this review to hospital materials, more research on materials used in hospital environments must be done to identify the composition and relevant manufacturing or post-manufacturing processes that could affect materials’ response to UV-C exposure.

The critical role of distance emerged across studies, following inverse-square law dynamics. PVC’s cytotoxic threshold at ≤50 cm^14^ and polycarbonate’s 39% slower degradation at 1.5 m^20^ suggest hospitals should establish minimum safe distances for sensitive equipment. These findings compel reconsideration of UV-C device placement, particularly for mobile units operating <1 m from surfaces. Future standards should incorporate distance-specific thresholds alongside time/dose parameters, especially for polymers like PLA and PVC where proximity dramatically alters degradation kinetics.

Material properties, including molecular structure, composition, and surface treatment, played a significant role in the degree of degradation observed.^25^ In general, more transparent and lightweight plastics such as polycarbonate were particularly prone to both visual and structural damage.^12,17,20^ Common surface changes including yellowing, loss of transparency, cracking, and the development of microfractures.^12,17,20^ While color change may appear cosmetic, its occurrence was often associated with deeper structural changes and loss of mechanical integrity.^17^

A wide range of methodologies were employed to detect and characterize material degradation, with varying degrees of sensitivity and specificity.^17,19^ Visual inspection and microscopy (optical and scanning electron) were commonly used for detecting color changes and surface cracks.^20,24^ While visual inspection remains the most practical method for routine monitoring in healthcare settings, detecting obvious changes like yellowing in polycarbonate or surface cracks in PLA,^20,24^ its limitations are well-documented. The subjective nature of visual assessment leads to inter-observer variability, and more importantly, it often fails to identify early-stage molecular degradation before mechanical properties are compromised.^17,22^ Molecular alterations were identified using infrared spectroscopy, which revealed bond scission and polymer chain reorganization.^14,16,18–20,22^ In addition, nanoindentation techniques quantified changes in hardness, further supporting evidence of material embrittlement or softening post-exposure.^22^ These methods offer a multifaceted approach to monitoring degradation, and their integration could enhance preventive maintenance programs in healthcare facilities. For comprehensive monitoring, we recommend a tiered approach: routine visual inspections could be supplemented with periodic instrumental analyses—infrared spectroscopy for molecular changes^14,16,18–20,22^ and nanoindentation for hardness measurements^22—^particularly when materials approach critical exposure thresholds identified in our review (eg, after 24 h cumulative UV-C for PLA^24^). This combined methodology would balance practicality with detection accuracy, enabling healthcare facilities to implement standardized degradation assessment protocols that account for both visible changes and underlying material integrity.

Another important finding in a subset of studies was the potential of mitigation strategies, particularly chemical additives such as UV stabilizers and antioxidants.^12,18^ These interventions showed promise in slowing or preventing degradation in polymers like polycarbonate and PLA.^12,18^ Incorporating such protective additives may improve the resilience of materials routinely exposed to UV-C, offering a potential path forward in material selection and procurement.

As noted above, one limitation of our review is that included articles are all experimental research,^11–24^ and thus the data contained in the review articles does not represent the real world of hospital environments and the actual processes of UV-C exposure of materials in hospital environments. Furthermore, a formal assessment of risk of bias was not conducted. This is consistent with the methodological framework of scoping reviews, which aims to map the available evidence rather than evaluate study quality. Additionally, there is currently no validated quality assessment tool specifically suited for experimental studies conducted in laboratory settings, further justifying the absence of a risk of bias appraisal in this review. Reviewed papers identify changes as a result of UV-C exposure to materials that may occur in items potentially found in hospitals.^11–24^ However, it is not clear exactly how these experimental study results carry over to materials in actual hospital environments. Similarly, exposures described may not be similar to typical UV-C healthcare exposures, and those themselves may vary depending on area within the hospital and by the hospital depending on policies, resources, and practices.^26^ The combinations of materials in hospitals may vary. Additionally, from the set of included papers, it is not possible to identify potential variation in degradation that may occur as a combination of UV-C exposure and other disinfection processes also used in hospital settings. Therefore, more research needs to be done. All included articles were experimental designs carried out in laboratories rather than field environments. This allowed for controlled conditions that could isolate the effects of UV-C radiation on various materials. While field studies could provide additional insights into real-world degradation, the laboratory settings of these studies were important to understanding the fundamental process of material degradation under UV-C exposure.

This review highlights that UV-C radiation, while effective for disinfection, poses a material degradation risk, particularly for polymeric substances commonly used in healthcare infrastructure. The degradation is material-specific, dose-dependent, and progressive over time. Future research should focus on standardizing assessment methods, quantifying damage thresholds, and developing UV-resistant materials or coatings. Understanding the trade-offs between disinfection efficacy and material durability is crucial for optimizing both patient safety and facility maintenance.

## Supporting information

10.1017/ash.2025.10114.sm001 Suh et al. supplementary material Suh et al. supplementary material

## Supplementary material

The supplementary material for this article can be found at https://doi.org/10.1017/ash.2025.10114.
