# Supplementary material for: Impact of UV-C on material degradation: a scoping literature review
Source: Antimicrob Steward Healthc Epidemiol. 2025 Sep 2;5(1):e199. doi: 10.1017/ash.2025.10114 (PMC12415791; doi:10.1017/ash.2025.10114)
Supplement: Suh et al. supplementary material [file S2732494X25101149sup001.docx]

**Supplementary Appendix 1.** Search terms and strategies

**PubMed 8/30/24**

("UV device"[Title/Abstract:~6] OR "UV equipment"[Title/Abstract:~6] OR "UV devices"[Title/Abstract:~6] OR "UV plastic"[Title/Abstract:~6] OR "UV plastics"[Title/Abstract:~6] OR "UV microplastic"[Title/Abstract:~4] OR “UV microplastics” [Title/Abstract:~6] OR "UV nanoplastics"[Title/Abstract:~6] OR "UV nanoplastic"[Title/Abstract:~6] OR"UV polymer"[Title/Abstract:~6] OR "UV material"[Title/Abstract:~6] OR"UV wood"[Title/Abstract:~6] OR"UV metal"[Title/Abstract:~6] OR"UV metals"[Title/Abstract:~6] OR"UV metals"[Title/Abstract:~6] OR"UV metals"[Title/Abstract:~6] OR "UV metals"[Title/Abstract:~6] OR "UV fiber"[Title/Abstract:~6] OR "UV glass"[Title/Abstract:~6] OR "UV carbon nanotube"[Title/Abstract:~6] OR "UV fabric"[Title/Abstract:~6] OR "UV furniture"[Title/Abstract:~6] OR "UV bed"[Title/Abstract:~6] OR "UV beds"[Title/Abstract:~6] OR "UV ventilators"[Title/Abstract:~6] OR "UV ventilator"[Title/Abstract:~6] OR "UV respirator"[Title/Abstract:~6] OR "UV respirators"[Title/Abstract:~6] OR "UV endoscope"[Title/Abstract:~6] OR "UV endoscopes"[Title/Abstract:~6] OR "UV otoscope"[Title/Abstract:~6] OR "UV otoscopes"[Title/Abstract:~6] OR "UV mask"[Title/Abstract:~6] OR "UV masks"[Title/Abstract:~6] OR "UVC device"[Title/Abstract:~6] OR "UVC equipment"[Title/Abstract:~6] OR "UVC devices"[Title/Abstract:~6] OR "UVC plastic"[Title/Abstract:~6] OR "UVC plastics"[Title/Abstract:~6] OR "UVC microplastic"[Title/Abstract:~6] OR "UVC nanoplastics"[Title/Abstract:~6] OR "UVC nanoplastic"[Title/Abstract:~6] OR"UVC polymer"[Title/Abstract:~6] OR "UVC material"[Title/Abstract:~6] OR"UVC wood"[Title/Abstract:~6] OR"UVC metal"[Title/Abstract:~6] OR"UVC metals"[Title/Abstract:~6] OR"UVC metals"[Title/Abstract:~6] OR"UVC metals"[Title/Abstract:~6] OR "UVC metals"[Title/Abstract:~6] OR "UVC fiber"[Title/Abstract:~6] OR "UVC glass"[Title/Abstract:~6] OR "UVC carbon nanotube"[Title/Abstract:~6] OR "UVC fabric"[Title/Abstract:~6] OR "UVC furniture"[Title/Abstract:~6] OR "UVC bed"[Title/Abstract:~6] OR "UVC beds"[Title/Abstract:~6] OR "UVC ventilators"[Title/Abstract:~6] OR "UVC ventilator"[Title/Abstract:~6] OR "UVC respirator"[Title/Abstract:~6] OR "UVC respirators"[Title/Abstract:~6] OR "UVC endoscope"[Title/Abstract:~6] OR "UVC endoscopes"[Title/Abstract:~6] OR "UVC otoscope"[Title/Abstract:~6] OR "UVC otoscopes"[Title/Abstract:~6] OR "UVC mask"[Title/Abstract:~6] OR "UVC masks"[Title/Abstract:~6] OR "ULTRAVIOLET equipment"[Title/Abstract:~6] OR "ULTRAVIOLET devices"[Title/Abstract:~6] OR "ULTRAVIOLET plastic"[Title/Abstract:~6] OR "ULTRAVIOLET plastics"[Title/Abstract:~6] OR "ULTRAVIOLET microplastic"[Title/Abstract:~6] OR "ULTRAVIOLET nanoplastics"[Title/Abstract:~6] OR "ULTRAVIOLET nanoplastic"[Title/Abstract:~6] OR"ULTRAVIOLET polymer"[Title/Abstract:~6] OR "ULTRAVIOLET material"[Title/Abstract:~6] OR"ULTRAVIOLET wood"[Title/Abstract:~6] OR"ULTRAVIOLET metal"[Title/Abstract:~6] OR"ULTRAVIOLET metals"[Title/Abstract:~6] OR"ULTRAVIOLET metals"[Title/Abstract:~6] OR"ULTRAVIOLET metals"[Title/Abstract:~6] OR "ULTRAVIOLET metals"[Title/Abstract:~6] OR "ULTRAVIOLET fiber"[Title/Abstract:~6] OR "ULTRAVIOLET glass"[Title/Abstract:~6] OR "ULTRAVIOLET carbon nanotube"[Title/Abstract:~6] OR "ULTRAVIOLET fabric"[Title/Abstract:~6] OR "ULTRAVIOLET furniture"[Title/Abstract:~6] OR "ULTRAVIOLET bed"[Title/Abstract:~6] OR "ULTRAVIOLET beds"[Title/Abstract:~6] OR "ULTRAVIOLET ventilators"[Title/Abstract:~6] OR "ULTRAVIOLET ventilator"[Title/Abstract:~6] OR "ULTRAVIOLET respirator"[Title/Abstract:~6] OR "ULTRAVIOLET respirators"[Title/Abstract:~6] OR "ULTRAVIOLET endoscope"[Title/Abstract:~6] OR "ULTRAVIOLET endoscopes"[Title/Abstract:~6] OR "ULTRAVIOLET otoscope"[Title/Abstract:~6] OR "ULTRAVIOLET otoscopes"[Title/Abstract:~6] OR "ULTRAVIOLET mask"[Title/Abstract:~6] OR "ULTRAVIOLET masks"[Title/Abstract:~6]) AND ("Equipment Reuse"[Mesh] OR "Materials Testing"[Mesh] OR "Equipment Failure"[Mesh] OR Degradation[Title/Abstract] OR Damage*[Title/Abstract] OR Reuse[Title/Abstract] OR Reusability[Title/Abstract] OR Failure[Title/Abstract] OR Integrity[Title/Abstract] OR effect [Title] OR Structure[Title/Abstract] OR Resilience[Title] OR Aging[Title] OR Weathering[Title] OR Propert*[Title] OR Characteristic*[Title] OR Photodegradation[Title/Abstract] OR performance [Title/Abstract]

OR behavior[Title])

#2

"Equipment and Supplies"[Mesh] AND "Ultraviolet Rays"[MeSH] AND ("Equipment Reuse"[Mesh] OR "Materials Testing"[Mesh] OR "Equipment Failure"[Mesh])

#3

(germicidal irradiation[Title/Abstract] OR germicidal light[Title/Abstract] OR sterilizing radiation[Title/Abstract]) AND ("Equipment Reuse"[Mesh] OR "Materials Testing"[Mesh] OR "Equipment Failure"[Mesh] OR Degradation[Title/Abstract] OR Damage*[Title/Abstract] OR Reuse[Title/Abstract] OR Reusability[Title/Abstract] OR Failure[Title/Abstract] OR Integrity[Title/Abstract] OR effect [Title/Abstract] OR Structure[Title/Abstract] OR Resilience[Title/Abstract] OR Aging[Title/Abstract] OR Weathering[Title/Abstract] OR Propert*[Title/Abstract] OR Characteristic*[Title/Abstract] OR Photodegradation[Title/Abstract]) AND ("Equipment and Supplies"[Mesh] OR device[Title/Abstract] OR equipment[Title/Abstract] OR plastic*[Title/Abstract] OR Microplastic*[Title/Abstract] OR Nanoplastic*[Title/Abstract] OR Poly*[Title/Abstract] OR Material*[Title/Abstract] OR Wood[Title/Abstract] OR Metal*[Title/Abstract] OR Fiber*[Title/Abstract] OR Cloth*[Title/Abstract] OR Fabric*[Title/Abstract] OR Furniture[Title/Abstract] OR Glass[Title/Abstract] OR Carbon nanotube*[Title/Abstract])

#4

"Water"[Mesh] OR "Milk"[Mesh] OR "Air"[Mesh] OR "Review" [Publication Type] OR review [Title] OR "Comment" [Publication Type] OR "Editorial" [Publication Type] OR Preprint [Publication Type] OR "Neoplasms"[Mesh] OR "Drug Therapy"[Mesh] OR "drug therapy" [Subheading] OR "DNA"[Mesh] OR “Models, Animal"[Mesh] OR aquatic[Title] OR liquid[Title] OR milk[Title] OR oil [Title] OR oils [Title] OR fluid* [Title] OR Wastewater[Title] OR sea water[Title] OR detector[Title] OR gas[Title] OR fabrication [Title] OR pesticide*[Title] OR semiconductor* [Title] OR in vitro[Title] OR diode* [Title] OR telescope*[Title] OR ink [Title] OR lithography[Title] OR Biofilm[Title] OR Electron accumulation[Title] OR laser*[Title] OR vapor*[Title] OR skin [Title] OR endothelium[Title] OR cancer*[Title] OR therap*[Title] OR outdoor [Title] OR photodetector* [Title] OR freshwater [Title] OR marine [Title] OR fish* [Title] OR "Fishes"[Mesh] OR semiconductor* [Title] OR biofilm* [Title] OR drug* [Title] OR cell* [Title] OR antimicrobial [Title] OR protocol [Title]

(#1 OR #2 OR #3) NOT #4=2426, with limit for English language applied

**Web of Science 8/30/24**

#1

TI=((UV NEAR/4 *degradation) OR (UVC NEAR/4 degradation) OR (ultraviolet NEAR/4 degradation) OR ("germicidal irradiation" NEAR/4 degradation) OR ("germicidal light" NEAR/4 degradation) OR (UV NEAR/4 damage*) OR (UVC NEAR/4 damage*) OR (ultraviolet NEAR/4 damage*) OR ("germicidal irradiation" NEAR/4 damage*) OR ("germicidal light" NEAR/4 damage*) OR (UV NEAR/4 Reuse) OR (UVC NEAR/4 Reuse) OR (ultraviolet NEAR/4 Reuse) OR ("germicidal irradiation" NEAR/4 Reuse) OR ("germicidal light" NEAR/4 Reuse) OR (UV NEAR/4 Reusability) OR (UVC NEAR/4 Reusability) OR (ultraviolet NEAR/4 Reusability) OR ("germicidal irradiation" NEAR/4 Reusability) OR ("germicidal light" NEAR/4 Reusability) OR (UV NEAR/4 Failure) OR (UVC NEAR/4 Failure) OR (ultraviolet NEAR/4 Failure) OR ("germicidal irradiation" NEAR/4 Failure) OR ("germicidal light" NEAR/4 Failure) OR (UV NEAR/4 structure*) OR (UVC NEAR/4 structure*) OR (ultraviolet NEAR/4 structure*) OR ("germicidal irradiation" NEAR/4 structure*) OR ("germicidal light" NEAR/4 structure*) OR (UV NEAR/4 resilience) OR (UVC NEAR/4 resilience ) OR (ultraviolet NEAR/4 resilience) OR ("germicidal irradiation" NEAR/4 resilience) OR ("germicidal light" NEAR/4 resilience) OR (UV NEAR/4 effect*) OR (UVC NEAR/4 effect*) OR (ultraviolet NEAR/4 effect*) OR ("germicidal irradiation" NEAR/4 effect*) OR ("germicidal light" NEAR/4 effect*) OR (UV NEAR/4 aging) OR (UVC NEAR/4 aging) OR (ultraviolet NEAR/4 aging) OR ("germicidal irradiation" NEAR/4 aging) OR ("germicidal light" NEAR/4 aging) OR (UV NEAR/4 weathering ) OR (UVC NEAR/4 weathering) OR (ultraviolet NEAR/4 weathering) OR ("germicidal irradiation" NEAR/4 weathering) OR ("germicidal light" NEAR/4 weathering) OR (UV NEAR/4 propert*) OR (UVC NEAR/4 propert*) OR (ultraviolet NEAR/4 propert*) OR ("germicidal irradiation" NEAR/4 propert*) OR ("germicidal light" NEAR/4 propert*) OR (UV NEAR/4 characteristic*) OR (UVC NEAR/4 characteristic*) OR (ultraviolet NEAR/4 characteristic*) OR ("germicidal irradiation" NEAR/4 characteristic*) OR ("germicidal light" NEAR/4 characteristic*))

#2

TI= (device* OR equipment OR *plastic* OR *Poly* OR Material* OR Wood OR Metal* OR Fiber* OR Cloth* OR Fabric* OR Furniture OR Glass OR Carbon nanotube*)

#3

TI= (review OR neoplasms* OR Therap* OR DNA OR “animal model" OR aquatic OR liquid* OR milk OR oil OR oils OR fluid* OR Wastewater OR sea water OR detector OR gas OR fabrication OR pesticide* OR semiconductor* OR in vitro OR diode* OR telescope* OR ink OR lithography OR Biofilm OR Electron accumulation OR laser* OR vapor* OR skin OR endothelium OR cancer* OR outdoor OR photodetector* OR freshwater OR marine OR fish* OR semiconductor* OR biofilm OR drug* OR cell* OR antimicrobial OR protocol*)

#1 AND #2 NOT #3=2194

with exclude filter for proceedings, meeting abstracts, review article, book chapter, news items, note, letters and restricted to English language

**Embase 8/30/24**

#1

'Ultraviolet Rays'/exp AND ('Equipment Reuse'/exp OR 'Materials Testing'/exp OR 'Equipment Failure'/exp)

#2

('germicidal irradiation':ti,ab OR 'germicidal light':ti,ab OR 'sterilizing radiation':ti,ab) AND ('Equipment Reuse'/exp OR 'Materials Testing'/exp OR 'Equipment Failure'/exp OR Degradation:ti,ab OR Damage*:ti,ab OR Reuse:ti,ab OR Reusability:ti,ab OR Failure:ti,ab OR Integrity:ti,ab OR effect:ti,ab OR Structure:ti,ab OR Resilience:ti,ab OR Aging:ti,ab OR Weathering:ti,ab OR Propert*:ti,ab OR Characteristic*:ti,ab OR Photodegradation:ti,ab) AND ('Equipment and Supplies'/exp OR device:ti,ab OR equipment:ti,ab OR plastic*:ti,ab OR Microplastic*:ti,ab OR Nanoplastic*:ti,ab OR Poly*:ti,ab OR Material*:ti,ab OR Wood:ti,ab OR Metal*:ti,ab OR Fiber*:ti,ab OR Cloth*:ti,ab OR Fabric*:ti,ab OR Furniture:ti,ab OR Glass:ti,ab OR 'Carbon nanotube*':ti,ab)

#3

(uvc:ti OR ultraviolet:ti) AND (degradation:ti OR damage*:ti OR reuse:ti OR reusability:ti OR failure,ti OR integrity:ti OR effect:ti OR structure:ti OR resilience:ti OR aging:ti OR weathering:ti OR propert*:ti OR characteristic*:ti OR photodegradation:ti OR performance:ti OR behavior:ti)

#3

Water/exp OR Milk/exp OR Air/exp  OR review:ti OR Neoplasms/exp OR 'Drug Therapy'/exp OR DNA/exp OR 'Models,Animal'/exp OR 'chapter'/it OR 'conference abstract'/it OR 'conference paper'/it OR 'editorial'/it OR 'letter'/it OR 'note'/it OR 'preprint'/it OR 'review'/it OR aquatic:ti OR liquid:ti OR milk:ti OR oil:ti OR oils:ti OR fluid*:ti OR Wastewater:ti OR 'sea water':ti OR detector:ti OR gas:ti OR fabrication:ti OR pesticide*:ti OR semiconductor*:ti OR 'in vitro':ti OR diode*:ti OR telescope*:ti OR ink:ti OR lithography:ti OR Biofilm:ti OR 'Electron accumulation':ti OR laser*:ti OR vapor*:ti OR skin:ti OR endothelium:ti OR cancer*:ti OR therap*:ti OR outdoor:ti OR photodetector*:ti OR freshwater:ti OR marine:ti OR fish*:ti OR Fishes/exp OR semiconductor*:ti OR biofilm*:ti OR drug*:ti OR cell*:ti OR antimicrobial:ti OR protocol:ti

#1 OR #2 OR #3 NOT #4=2267, limited to English

**Cochrane 8/30/24**

#1 [mh "Ultraviolet Rays"] AND ([mh "Equipment Reuse"] OR [mh "Materials Testing"] OR [mh "Equipment Failure"])

#2 (("germicidal irradiation" OR "germicidal light" OR "sterilizing radiation" OR UVC OR ultraviolet)):ti,ab,kw (Word variations have been searched)

#3 (([mh "Equipment Reuse"] OR [mh "Materials Testing"] OR [mh "Equipment Failure"] OR Degradation OR Damage* OR Reuse OR Reusability OR Failure OR Integrity OR effect OR Structure OR Resilience OR Aging OR Weathering OR Propert* OR Characteristic* OR Photodegradation) AND ([mh "Equipment and Supplies"] OR device OR equipment OR plastic* OR Microplastic* OR Nanoplastic* OR Poly* OR Material* OR Wood OR Metal* OR Fiber* OR Cloth* OR Fabric* OR Furniture OR Glass OR ("Carbon" NEXT nanotube*))):ti,ab,kw (Word variations have been searched)

#4 #2 AND #3 891

#5 #1 OR #4 891

=878, limited to trials

**CINAHL 8/30/24**

#1

MH "Ultraviolet Rays+" AND (MH "Equipment Reuse+" OR MH "Materials Testing+" OR MH "Equipment Failure+")

#2

(TI "germicidal irradiation" OR AB "germicidal irradiation" OR TI "germicidal light" OR AB "germicidal light" OR TI "sterilizing radiation" OR AB "sterilizing radiation" OR TI UVC OR TI ultraviolet ) AND (MH "Equipment Reuse+" OR MH "Materials Testing+" OR MH "Equipment Failure+" OR TI Degradation OR AB Degradation OR TI Damage* OR AB Damage* OR TI Reuse OR AB Reuse OR TI Reusability OR AB Reusability OR TI Failure OR AB Failure OR TI Integrity OR AB Integrity OR TI effect OR AB effect OR TI Structure OR AB Structure OR TI Resilience OR AB Resilience OR TI Aging OR AB Aging OR TI Weathering OR AB Weathering OR TI Propert* OR AB Propert* OR TI Characteristic* OR AB Characteristic* OR TI Photodegradation OR AB Photodegradation) AND (MH "Equipment and Supplies+" OR TI device OR AB device OR TI equipment OR AB equipment OR TI plastic* OR AB plastic*OR TI Microplastic* OR AB Microplastic* OR TI Nanoplastic* OR AB Nanoplastic* OR TI Poly* OR AB Poly*OR TI Material* OR AB Material* OR TI Wood OR AB Wood OR TI Metal* OR AB Metal* OR TI Fiber* OR AB Fiber* OR TI Cloth* OR AB Cloth* OR TI Fabric* OR AB Fabric* OR TI Furniture OR AB Furniture OR TI Glass OR AB Glass OR TI "Carbon nanotube*" OR AB "Carbon nanotube*")

#3

MH Water+ OR MH Milk+ OR MH Air+ OR PT Review OR TI review OR PT Comment OR PT Editorial OR PT Preprint OR MH Neoplasms+ OR MH "Drug Therapy+"  OR MH DNA+ OR MH "Models, Animal+" OR TI aquatic OR TI liquid OR TI milk OR TI oil OR TI oils OR TI fluid* OR TI Wastewater OR TI "sea water" OR TI detector OR TI gas OR TI fabrication OR TI pesticide* OR TI semiconductor* OR TI "in vitro" OR TI diode* OR TI telescope* OR TI ink OR TI lithography OR TI Biofilm OR TI "Electron accumulation" OR TI laser* OR TI vapor* OR TI skin OR TI endothelium OR TI cancer* OR TI therap* OR TI outdoor OR TI photodetector* OR TI freshwater OR TI marine OR TI fish* OR MH Fishes+ OR TI semiconductor* OR TI biofilm* OR TI drug* OR TI cell* OR TI antimicrobial OR TI protocol OR PT “brief item” OR PT proceedings

#1 OR #2 NOT #3= 298, limited to English and peer reviewed journals
